# P03-011 - Differential for granulomatosis with polyangiitis

**DOI:** 10.1186/1546-0096-11-S1-A207

**Published:** 2013-11-08

**Authors:** SM Rawn, P Miettunen, H Schmeling

**Affiliations:** 1 Department of Medicine, Calgary, Canada; 2 Department of Pediatric Rheumatology, University of Calgary, Calgary, Canada

## Introduction

Granulomatosis with Polyangiitis (GPA) is a rare and potentially lethal vasculitis, which predominantly affects small vessels. GPA can present with palpable purpura, hemorrhagic macules and bullae similar to skin lesions found in the most common small vessel vasculitis in childhood, Henoch-Schoenlein Purpura (HSP), leading to diagnostic confusion.

## Case report

We report the case of a previously healthy 14-year-old girl who presented with fever, congestion and sinus pain in May 2012. Initially diagnosed with bacterial sinusitis, she received antibiotics, but experienced ongoing fatigue and malaise. Within weeks, she developed abdominal pain, hematuria, migratory arthritis, palpable purpura on her lower and upper extremities and was diagnosed with HSP. Two weeks later, she was admitted with hemoptysis, abnormal urinalysis, ischemic digits and progression of the rash into large bullae and ulcerations. Her renal biopsy revealed pauci-immune necrotizing glomerulonephritis, and her antineutrophil cytoplasmic antibodies and anti-proteinase 3 antibodies were high positive leading to a diagnosis of GPA. She received six months of treatment with oral cyclophosphamide and high dose intravenous and oral steroids. The skin lesions required debridement. Due to ongoing inflammation, with progression to nasal septal perforation, Rituximab, an anti-B-cell antibody, was initiated at month seven. This allowed her clinical picture and laboratory abnormalities to improve, with return to normal renal function.

## Discussion

In this case, a pediatric patient with GPA initially presented with the classic tetrad of HSP: palpable purpura, arthritis/arthralgias, abdominal pain and hematuria. This child subsequently developed hemoptysis and progression of her rash and ischemic digits prior to a diagnosis of GPA being made. In conclusion, if a patient with a severe case of HSP has extensive skin involvement, it is vital to consider a diagnosis of GPA to avoid serious organ or life threatening consequences.

## Competing interests

None Declared.
